# Risks of second non-breast primaries following breast cancer in women: a systematic review and meta-analysis

**DOI:** 10.1186/s13058-023-01610-x

**Published:** 2023-02-10

**Authors:** Isaac Allen, Hend Hassan, Eleni Sofianopoulou, Diana Eccles, Clare Turnbull, Marc Tischkowitz, Paul Pharoah, Antonis C. Antoniou

**Affiliations:** 1 Department of Public Health and Primary Care, University of Cambridge, Cambridge, UK; 2 Department of Cancer Sciences, Faculty of Medicine, University of Southampton, Southampton, UK; 3 Translational Genetics Team, Division of Genetics and Epidemiology, Institute of Cancer Research, London, UK; 4 Department of Medical Genetics, Cambridge Biomedical Research Centre, National Institute for Health Research, University of Cambridge, Cambridge, UK; 5 Centre for Cancer Genetic Epidemiology, University of Cambridge, Cambridge, CB1 8RN UK

**Keywords:** Breast neoplasms, Second primary, Second cancer, Multiple primary, Multiple cancer, Risk, Incidence, Epidemiology, Systematic review, Meta-analysis

## Abstract

**Background:**

Second primary cancer incidence is rising among breast cancer survivors. We examined the risks of non-breast second primaries, in combination and at specific cancer sites, through a systematic review and meta-analysis.

**Methods:**

We conducted a systematic search of PubMed, Embase, and Web of Science, seeking studies published by March 2022. We included studies that reported standardized incidence ratios (SIRs), with associated standard errors, assessing the combined risk of second non-breast primaries following breast cancer. We performed meta-analyses of combined second primary risks, stratifying by age, follow-up duration, and geographic region. We also assessed second primary risks at several specific sites, stratifying by age. The inverse variance method with DerSimonian–Laird estimators was used in all meta-analyses, assuming a random-effects model. Associated biases and study quality were evaluated using the Newcastle–Ottawa scale.

**Results:**

One prospective and twenty-seven retrospective cohort studies were identified. SIRs for second non-breast primaries combined ranged from 0.84 to 1.84. The summary SIR estimate was 1.24 (95% CI 1.14–1.36, *I*^2^: 99%). This varied by age: the estimate was 1.59 (95% CI 1.36–1.85) when breast cancer was diagnosed before age 50, which was significantly higher than in women first diagnosed at 50 or over (SIR: 1.13, 95% CI 1.01–1.36, *p* for difference: < 0.001). SPC risks were also significantly higher when based on Asian, rather than European, registries (Asia—SIR: 1.47, 95% CI 1.29–1.67. Europe—SIR: 1.16, 95% CI 1.04–1.28). There were significantly increased risks of second thyroid (SIR: 1.89, 95% CI 1.49–2.38), corpus uteri (SIR: 1.84, 95% CI 1.53–2.23), ovary (SIR: 1.53, 95% CI 1.35–1.73), kidney (SIR: 1.43, 95% CI 1.17–1.73), oesophagus (SIR: 1.39, 95% CI 1.26–1.55), skin (melanoma) (SIR: 1.34, 95% CI 1.18–1.52), blood (leukaemia) (SIR: 1.30, 95% CI 1.17–1.45), lung (SIR: 1.25, 95% CI 1.03–1.51), stomach (SIR: 1.23, 95% CI 1.12–1.36) and bladder (SIR: 1.15, 95% CI 1.05–1.26) primaries.

**Conclusions:**

Breast cancer survivors are at significantly increased risk of second primaries at many sites. Risks are higher for those diagnosed with breast cancer before age 50 and in Asian breast cancer survivors compared to European breast cancer survivors. This study is limited by a lack of data on potentially confounding variables. The conclusions may inform clinical management decisions following breast cancer, although specific clinical recommendations lie outside the scope of this review.

**Supplementary Information:**

The online version contains supplementary material available at 10.1186/s13058-023-01610-x.

## Background

Multiple studies have compared the risk of second primary cancers (SPCs) following a first breast cancer (BC) to the corresponding first cancer risks in the general population [1–33]. Although most of these studies report an elevated risk [1, 2, 4–6, 8–33], the magnitudes of the reported associations vary widely. Since a 2015 review reported a 17% increase in SPC risks following BC [34], many new studies have been published [1, 5, 6, 9, 12, 16, 17, 19, 20, 23, 24, 27, 32]. In addition, BC is both increasing in incidence and improving in survival outcomes [35–37], exacerbating the public health problem posed by SPCs in BC survivors. Updated pooled estimates of SPC risks following BC are hence due.

Most published studies to date drew their data from European or North American population-based cancer registries [1–17, 28–31, 33], although several also drew their data from Asian registries [18–27, 32]. Many studies have found BC survivors to be at increased risk of melanoma [1, 7, 13, 14, 29–31, 33], thyroid cancer [1, 15, 19, 20, 23–25, 27, 29–31, 33, 38], and several cancers of the urogenital and gastrointestinal systems [1, 2, 4, 6–33], although the estimated magnitude of these risks varies.

A systematic review of the latest published evidence on SPC risks is helpful in guiding clinical management following BC. This could lead to improvements in SPC prevention and early detection.

In this review, we examine the latest evidence regarding the combined risks of developing SPCs following a first primary BC. We also evaluate the variability in SPC risks caused by confounding variables such as patient characteristics and demographic information. Finally, we identify which cancer sites may drive the combined risk of SPCs and quantify the magnitude of these site-specific risks.

## Methods

### Exposure, outcome and measures of association

The exposure was the diagnosis of a primary BC. The outcome was the later diagnosis of a non-breast SPC. The measure of association was the standardized incidence ratio (SIR) comparing the incidence of second non-breast primaries among BC survivors to the incidence of first non-breast primaries in the general population.

To ensure the review accurately assessed second primary risks, a key condition of inclusion was that a study should have made a clear effort to differentiate SPCs from recurrences or metastatic developments of the first primary BC. For example, guidance on the topic is provided by the Surveillance, Epidemiology and End Results (SEER) programme [39]. Separate guidelines are also provided by the International Association of Cancer Registries (IACR)/International Agency for Research on Cancer (IARC) [40, 41]. However, a study by Coyte et al. found counts of second *breast* primaries following a first BC to differ between the SEER and IARC/IACR guidelines and counts of all other primaries to agree very closely [42]. Since the SEER guidelines entail standard practice in North America and the IARC/IACR guidelines entail standard practice in all other areas, it was anticipated that most studies would use these guidelines, and therefore that we would have been unable to draw meaningful conclusions about second primary BC risk. As a result, only second non-breast cancers were considered as an outcome in this review. To make use of more data, we did not restrict on the types of efforts to differentiate SPCs from recurrences or metastases that studies made.

### Data sources and search strategy

Embase, PubMed, and Web of Science were searched on 11th March 2022 using the below queries:

#### Embase

(Breast Neoplasms/ or “breast cancer”) and (Neoplasms, Second Primary/ or “second cancer” or “second primary”) and risk

#### PubMed

(“Breast Neoplasms”[MeSH] OR “breast cancer”) AND (“Neoplasms, Second Primary”[MeSH] OR “second cancer” OR “second primary”) AND risk

#### Web of science

(TS = ((“breast cancer” OR “breast neoplasm”) AND (“second cancer” or “second primary”) AND risk)) OR (AB = ((“breast cancer” OR “breast neoplasm”) AND (“second cancer” or “second primary”) AND risk))

### Inclusion and exclusion criteria

To be included in the review, a study had to provide all information needed to extract a SIR and associated standard error evaluating the combined risk of non-breast SPCs in female BC survivors. It also had to take clearly described steps to discern SPCs from recurrences or metastases of the first BC, use data predominantly on those aged 15 and above at BC diagnosis, and be written in English.

A study would be excluded if it evaluated SPC risks only in survivors of a non-invasive BC or only following a specific treatment of the first BC. Studies would also be excluded if data on third or subsequent primaries could not be excluded from their SPC risk estimates or if their data overlapped entirely with another accepted study.

Studies with data that partly but not fully overlapped were included in the review. In this case, the study with a greater sample size was the only one included in any meta-analyses. If this could not be established, the study including the most recent data was the one included.

There is a particularly close data link between the Swedish Family Cancer Database and the Swedish national cancer registry [43]. The same is true of the Taiwanese Registry of Catastrophic Illness and the national cancer registry of Taiwan [44]. We therefore considered data from these centres to overlap. Similarly, data from the Osaka Medical Centre for Cancer and Cardiovascular Diseases (OMCC) are primarily a subset of Osaka Cancer Registry (OCR) data [45]. Accordingly, if a study based on OMCC data overlapped with a study based on OCR data, the latter was considered the larger study if there was missing information on sample size.

### Data extraction

Title and abstract screening was performed by two authors as part of an independent double-screening process. Conflicts regarding twelve studies were resolved by another author. We closely read the full text, swept the bibliographies, and whenever applicable searched the PubMed “cited by” sections of each the studies that passed the title and abstract screening in search of additional studies.

### Statistical analysis

We assumed there would be some between-study variance in SIRs not attributable to sampling error, and therefore assumed a random-effects model in all meta-analyses [46], using the generic inverse variance method with DerSimonian–Laird estimators [47, 48]. Standard errors were extracted routinely [49] and were used to weight the studies in meta-analyses [46]. We used Byar’s approximation to calculate confidence intervals (CIs), unless CIs could be taken directly from a study [49].

We firstly performed an unstratified meta-analysis. We quantified the heterogeneity (variation in true effect sizes between studies [46, 47]) in these results by inspecting Cochran’s *Q* [48] and the *I*^2^ statistic [50, 51]. Cochran’s *Q* is the sum of squared differences between the estimate of the pooled effect size and the effect sizes reported by each study, weighted by the inverse variances of the studies [46]. The *I*^2^ statistic is the percentage by which the observed value of Cochran’s *Q* exceeds the value expected under the null hypothesis of no between-study heterogeneity [46].

We also performed leave-one-out analyses to identify which studies were the main drivers of heterogeneity [46], which we defined as the studies causing Cochran’s *Q* to decrease by over 10% once they were removed from the unstratified meta-analysis. We also defined outlier studies to be studies which reported SIRs with 95% confidence intervals that lay wholly outside the confidence interval around the summary SIR generated by the unstratified meta-analysis [46]. We then performed two further meta-analyses after, respectively, eliminating all the main drivers of heterogeneity and all outlier studies, to assess the remaining heterogeneity and the effect on the summary SIR. We examined publication bias by visually assessing funnel plots and performing Egger’s test [52].

We also performed further meta-analyses stratifying on (1) age at BC diagnosis—under 50 years and 50 years or above. Data on those diagnosed before age 56 and at age 56 or over were, respectively, included in the younger and older strata if no stratification at 50 was provided, (2) follow-up time duration following BC diagnosis—under 5 years or 5 years and over. We also performed a second meta-analysis stratifying at 10 years, (3) geographic region—the continent of the data centre (i.e., hospital, registry) used in a particular study.

We evaluated for differences in risks by age, follow-up duration, and geographic region using the Cochran’s *Q* statistic, by considering each stratum as a subgroup, and by comparing the resulting statistic to a chi-squared distribution [46].

We also examined the Cochran’s *Q* and *I*^2^ statistics in each stratum for each stratified meta-analysis, to assess if a particular risk factor explained some of the heterogeneity in the unstratified analysis of non-breast SPC risks.

We extracted SIRs that quantified SPC risks at specific sites, together with associated standard errors, from the studies included in the unstratified meta-analysis. We then estimated summary SIRs for SPC risks at these sites by conducting meta-analyses of the relevant site-specific SIRs. This was done to elucidate which cancer sites were driving the combined risks of all non-breast SPCs. We first examined site-specific risks for all ages. We then stratified by age at BC diagnosis, using the same stratification points as in the analyses of combined non-breast primary risks. These analyses were performed for each of the 20 non-breast cancer sites with the highest incidence among women worldwide in 2020, excluding non-melanoma skin cancer and excluding oral cavity and lip cancer due to SPC risks at this site often being combined with other head and neck sites [6, 23, 33]. These sites are the bladder, the blood (leukaemia, myeloma, and non-Hodgkin’s lymphoma), the brain and central nervous system (CNS), the cervix uteri, the corpus uteri, the colorectum, the gallbladder, the kidney, the liver, the lung, the oesophagus, the ovary, the pancreas, the skin (melanoma), the stomach, the thyroid, and the vulva [53].

Forest plots were generated as a visual aid to accompany each meta-analysis. We evaluated the methodological quality of each study using the Newcastle–Ottawa scale (NOS) [54], as recommended by the Cochrane Collaboration [47] (details in Additional file 1). RStudio version 4.1.2 was used for all analyses [55]. We defined statistical significance to be present when a *p* value of under 0.05 was observed.

## Results

### Results of literature search

**Fig. 1:**
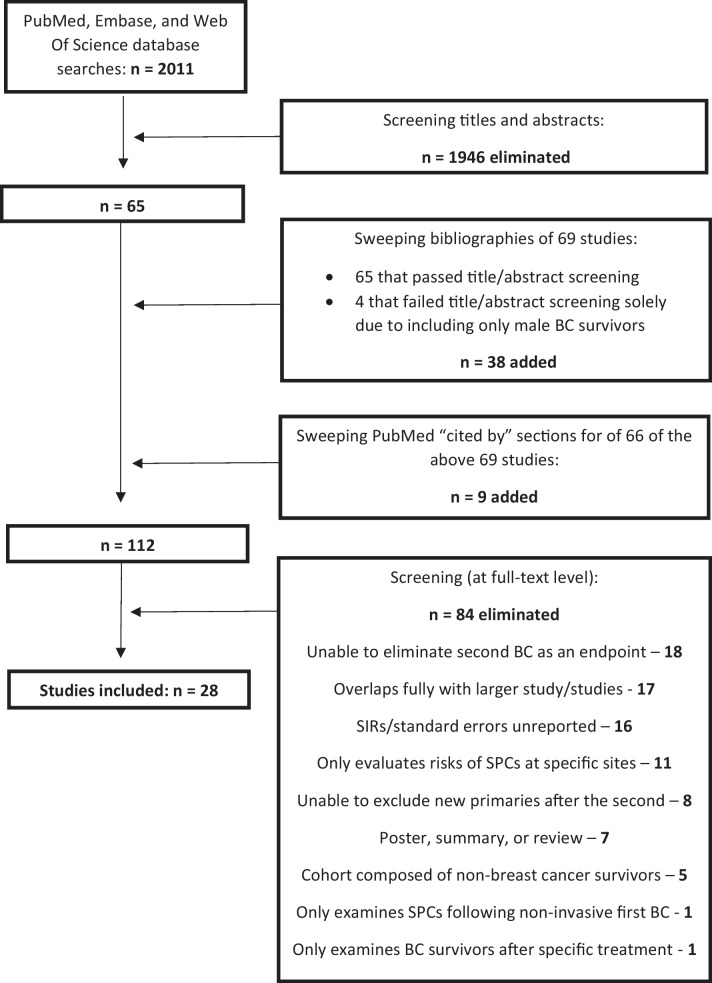
Search process

In total, 112 studies were accepted for review at the full-text level after passing the title and abstract screening stage. Sixty-five of these were selected from the 2011 studies returned after the database searches. Thirty-eight of the 112 studies were found following sweeps of the bibliographies of 69 studies: the 65 studies previously mentioned, and 4 additional studies which only failed the title and abstract sweeping due to exclusively examining male BC survivors. We identified the final 9 of the 112 studies after sweeping the “cited by” section of PubMed for 66 of these 69 studies, as the remaining three studies [56–58] were unavailable in PubMed. In this way, we hoped to capture additional relevant literature published both before and after the studies identified through the database searches. Following close reading, we included 28 of the 112 studies in this review. Reasons for exclusions of the remaining 84 studies, as well as a full explanation of the search process, are shown in Fig. 1.

All studies included were cohort studies, only one of which was prospective [12]. Three studies were hospital-based [13, 15, 20], and the remainder were wholly or predominantly registry-based. The centre/centres (hospital or registry/registries) were European in fourteen studies [1–5, 7, 9–14, 16, 17], Asian in ten studies [18–27], and North American in three studies [6, 8, 15]. One study [33] drew their cohort from registries based across four continents. Since the bulk of the cohort was taken from European registries, this study was treated as European for the purposes of any stratifications based on geographic region. Three [4, 5, 12] studies used data from multiple countries in Europe, although all the data drawn from non-German centres in Chen et al. [5] fully overlapped with larger studies [17, 33]. Therefore, we only included the German data from Chen et al. in this review.

The longest follow-up period was 57 years [17]. The shortest was 11 years [12, 26].

Six studies set minimum ages at first cancer diagnosis, at age 15 years [5, 11, 16, 23] and age 20 years [18, 20]. Six studies set maximum ages: at age 39 years [16], age 79 years [23, 25, 26], age 84 years [7], and age 89 years [2]. The used cohort in one study [12] was taken from a pre-existing larger observational cohort study. The original larger cohort included participants between ages 35 years and 70 years at recruitment without regard to cancer status. The subset of the participants from this larger cohort who subsequently developed a first primary BC formed the cohort included in this review. All remaining nineteen studies imposed no age-related restrictions when selecting their cohorts.

Fifteen studies excluded data on second primaries occurring within some given follow-up duration following the first BC diagnosis [2–4, 8–11, 13, 18, 20–23, 25, 26]. All other studies included data on second primaries diagnosed immediately following the first BC, although the study by the AIRTUM Working Group [1] also gave a separate analysis excluding SPCs diagnosed in the first 2 months of follow-up. The data excluding the earlier SPCs were explicitly stated as less prone to bias by the authors, so these were the data used in any statistical analyses.

All but one study [5] gave site-specific risks of second primaries.

The reported SIRs ranged from 0.84 [3] to 1.84 [23]. All but five [3, 7, 18, 20, 23] estimated SIRs ranging between 1.00 and 1.50.

The characteristics of all 28 studies are detailed in Table 1 and Table 2. The NOS scores assigned to each study may be seen in Additional file 1, together with an explanation of the methods used.

**Table 1:** Study characteristics

Author and publication year	Period of first BC^1^ dx^2^ for cohort	Follow-up period	Study design	Country and centre of data derivation	Definition of cohort	Definition of second primary cancers
AIRTUM Working Group [1]	Dx: 1976–2010 (varied by registry)	Start: At BC dx End: At SPC dx, death, date of last known vital status, or end of last year of registration (dates varied by registry)	Retrospective cohort	Italy (Multiple cancer registries covering 48% of the population)	All patients dx with a first cancer, although melanoma skin cancer cases, cases based on death certificate only, cases based on autopsy only, and cases with follow-up time equal to zero were excluded. Cohort was stratified by first cancer site, allowing analysis for first BC	IARC/IACR^4^ rules
Andersson [2]	Dx 1977–2001	Start: 1y after BC dx End: SPC dx, death, emigration, or study end (2002)	Retrospective cohort	Denmark (Danish Breast Cancer Cooperative Group)	Female BC patients with record of BC dx at under age 90 in both the Danish Breast Cancer Cooperative Group and the Danish Cancer Register, who survived at least 1y^5^ post-BC dx, with no prior cancer history other than non-melanoma skin cancer, treated and followed accorded to a Danish Breast Cancer Cooperative Group protocol	SPC^6^ coding rules unstated, but the Danish Breast Cancer Cooperative Group is linked to the Danish Cancer Register, which uses IARC/IACR rules
Brenner [3]	Dx 1968–1987	Start: 1y after BC dx End: study end given as 1987. No details of other censoring events provided	Retrospective cohort	Germany (Saarland Cancer Registry)	Women dx with a first BC (first 1y post dx excluded from analysis)	SPC coding rules unstated, but German registries use IARC/IACR rules. Secondary malignancies and tumours of unspecified location, the skin, the bone, the brain and nervous system, the lung and the liver were excluded
Brown [4]	Dx 1943–1999 (Denmark), 1953–2002 (Finland), 1953–2000 (Norway), 1958–2002 (Sweden)	Start: 1y after BC dx End: SPC dx, death, or study end (1999–2002, depending on registry)	Retrospective cohort	Denmark, Finland, Norway, Sweden (all national registries)	Women dx with a first BC, who survived for at least 1y (first 1y post dx excluded from analysis)	SPC coding rules unstated, but all participating registries use IARC/IACR rules. Non-haematological malignancies excluded
Chen [5]	Dx 1997–2010	Start: At BC dx End: SPC dx, death, emigration, or study end (2010)	Retrospective cohort	Germany (12 German cancer registries covering 33% of population). Data was also reported for Sweden, but is not included here due to fully overlapping with several larger studies	Patients aged 15y or over at dx of a first primary malignant tumour. Patients with only death certificate/autopsy information were excluded. Cohort was stratified by first cancer site, allowing analysis for first BC	According to IARC/IACR rules, not including non-melanoma skin cancer. All cancers must be discordant. 95% + of were cancers microscopically verified
Diab [6]	Dx 1973–2012	Start: At BC dx End: SPC dx, death, or study end (2015)	Retrospective cohort	The USA—9 SEER^7^ registries (Connecticut, Detroit, Atlanta, San Francisco (Oakland), Hawaii, Iowa, New Mexico, Seattle (Puget Sound), Utah)	Women dx with breast cancer. In situ malignancies, dx made without microscopic confirmation, and dx from death certificates and autopsy reports were not included	SEER rules
Evans [7]	Two cohorts pooled: Dx 1961–1970 and dx 1971–1995	Start: At BC dx End: SPC dx, death, loss to follow-up, 85th birthday, or study end (1982 for those dx 1961–70, 1996 for those dx 1971–1995)	Retrospective cohort	England (Thames Cancer Registry)	Women dx with first BC at under age 85	Second tumours at a separate anatomical site and of a distinct histological type to the first tumour, or stated as a new tumour by the treating clinician. Non-melanoma skin cancers, non-malignant cancers, second cancers occurring within 1y of the initial cancer at the same site with the same laterality and histology, or cancers in patients without residency information available at date of dx or a without given date of dx were all excluded
Gulhan [18]	1992–2006	Start: 1y after BC dx End: study end given as 2006. No details of other censoring events provided	Retrospective cohort	Turkey (Izmir Cancer Registry)	Women aged at least 20 with histologically confirmed invasive BC, with at least 1m^8^ of follow-up	IARC/IACR rules
Harvey [8]	1935–1982	Start: 2m after BC dx End: At SPC dx, death, date of last known vital status, or study end (1982)	Retrospective cohort	The USA (Connecticut Tumour Registry)	Individuals diagnosed with a first primary invasive BC when they were resident in Connecticut, that survived without a second cancer developing for at least 2 m after the diagnosis, who were observed for at least 2 m after the diagnosis, and whose cancer was not diagnosed only from an autopsy report or death certificate	Most of the data used in this study predates the publication of the SEER SPC coding rules, the most common rules applied in North America. However, SPC coding rules used in this study were very similar. Briefly, study defined SPCs as invasive cancers that developed at least 2 m after the first cancer, excluding in situ cancers or non-melanoma skin cancers. SPCs diagnosed only from autopsy reports or death certificates were included
Hung 2016 [19]	1997–2010	Start: At BC dx End: SPC dx, death, dropout from programme providing study data, or study end (2011)	Retrospective cohort	Taiwan (Registry of Catastrophic Illness)	Patients dx with a first BC	SPC coding rules unstated, but the registry histologically confirms cancer cases. Oncologists are required to give evidence of the diagnosis, including cytology reports, pathology reports, laboratory studies, and imaging studies, for review by commissioned expert panels
Jégu [9]	Dx 1989–2004	Start: 2 m (62 days) after BC dx End: At SPC dx, death, date of last known vital status, or study end (2007)	Retrospective cohort	France (10 registries covering the Bas-Rhin, Calvados, Doubs, Hérault, Isère, Manche, Somme and Tarn administrative regions)	Patients dx with a first cancer, who did not develop a SPC within 2 m (62 days) after their first cancer. Cohort was stratified by first cancer site, allowing analysis for first BC	IARC/IACR rules
Jung [20]	Dx 1989–2014	Start: At BC dx End: At SPC dx, death, date of last known hospital visit, or study end (2014)	Retrospective cohort	Korea (3 medical centres in Soeul, Bucheon, and Choenan)	Women aged at least 20y dx with BC and with at least 1 visit to the Soeul, Bucheon, or Choenan centres within 2 m from dx and with treatment records, who contributed at least 2 m of follow-up time	SPC coding rules unspecified, but second cancers must be at discordant sites, dx at least 2 m after BC diagnosis, with each case "thoroughly reviewed, and misleading information from breast cancer metastasis excluded”
Lee [21]	Dx 1979–2003	Start: At BC dx End: At SPC dx, death, or study end (2003)	Retrospective cohort	Taiwan (National Cancer Registry)	Women dx with first BC, without missing dates of birth, follow-up dates or death statuses, and who survived without a second cancer for at least 1 m post-BC dx	According to IARC/IACR rules. Second cancers reported within 1 m of BC dx excluded
Levi [10]	Dx 1974–1998	Start: At BC dx End: At SPC dx, death, emigration, or study end (1998)	Retrospective cohort	Switzerland (Swiss Cancer Registries of Vaud and Neuchâtel)	Women dx with a first BC with at least 1 m of follow-up	SPC rules unstated, but the Vaud and Neuchâtel registries use IARC/IACR rules. Second cancers diagnosed at autopsy, death, by death certification alone, or within 1 m of first BC were excluded. Second cancers must be morphologically different or at different anatomical sites
Mellemkjaer [33]	Australia, New South Wales: 1972–1997, Canada, British Colombia: 1970–1998, Canada, Manitoba: 1970–1998, Canada, Saskatchewan: 1967–1998, Denmark: 1943–1997, Finland: 1953–1998, Iceland: 1955–2000, Norway: 1953–1999, Singapore: 1968–1992, Slovenia: 1961–1998, Spain, Zaragoza: 1978–1998, Sweden: 1961–1998, UK, Scotland: 1960–1996	Start: At BC dx End: At SPC dx, death, emigration, or study end (between 1992 and 2000, depending on registry)	Retrospective cohort	13 large cancer registries. Canada (British Columbia, Manitoba and Saskatchewan), Singapore, Slovenia, Norway, Denmark, Scotland, Australia (New South Wales), Sweden, Finland, Iceland, Spain (Zaragoza)	Women dx with a first BC	IARC/IACR rules. Tumours identified by following the recording practices of the included registries
Molina-Montes [11]	Dx 1985–2007	Start: At BC dx End: At SPC dx, death, or study end (2007)	Retrospective cohort	Spain (Granada Cancer Registry)	Women dx with a first BC, aged 15y or over at BC dx	According to IARC/IACR rules. Second cancers only included if they occurred at least 3 m after the BC dx
Murakami [22]	Dx 1965–1982	Start: Unstated, but less than 1y after BC dx End: At SPC dx, death, or study end (1983)	Retrospective cohort	Japan (Osaka Cancer Registry)	Women dx with a first BC who survived at least 3 m after the BC dx	SPC rules unspecified but Osaka Cancer Registry follows IARC/IACR rules. Second cancers only included if they occurred at least 3 m after the BC dx
Odani [23]	2000–2014	Start: 3 m after BC dx End: At SPC dx, death, 10y after BC dx, or study end (2015)	Retrospective cohort	Japan (Osaka Cancer Registry)	Dx with first primary invasive cancer, aged 15–79 years and resident in Osaka at dx. Dx with death certificate only were excluded. Cohort was stratified by first cancer site, allowing analysis for first BC	IARC/IACR rules
Ricceri [12]	Individuals recruited to cohort of generally healthy individuals between 1992 and 1998. The subset of these that developed a first primary BC during 11y of follow-up was taken as the cohort of BC survivors in this study	Start: At BC dx End: at SPC dx, death, or end of study (year of study end unstated)	Prospective cohort	EPIC^9^ cohort is drawn from generally healthy individuals aged 35–70 from 23 centres from Denmark, France, Germany, Greece, Italy, the Netherlands, Norway, Spain, Sweden and the UK. Follow-up for cancer was based on population cancer registries except in France, Germany and Greece, where a combination of methods including health insurance records, cancer and pathology registries and active follow-up were used	Female subset of EPIC cohort that developed a first BC after recruitment into study, or that developed a BC as their second cancer after a first non-melanoma skin cancer. Cases identified using death certificate only were excluded	IARC/IACR rules. Second cancers dx on same date as initial BC or identified using death certificate only were excluded
Rubino [13]	1954–1984	Start: 1973, for those dx with BC 1954–1971. 1y after BC dx, for those dx with BC 1972–1984 End: At SPC dx, death, loss to follow-up, or study end (1992)	Retrospective cohort	France (Institut Gustave Roussy)	Women dx with first BC, born and living in France, with at least 1y of follow-up since BC dx	SPC rules unspecified. All second malignancies were histologically confirmed. Second bilateral BCs and non-melanoma skin cancers were excluded
Schaapveld [14]	Groningen and Amsterdam: 1989–2003 Eindhoven: 1989–2002	Start: At BC dx End: at SPC dx, death, or end of study (Groningen and Amsterdam: 2005. Eindhoven: 2004)	Retrospective cohort	The Netherlands (Comprehensive cancer centres of Groningen, Amsterdam, and Eindhoven)	Women dx with first BC with no prior cancer history, or a first BC following non-melanoma skin cancer	According to IARC/IACR coding rules. All unknown primary adenocarcinomas, meningiomas, myelodysplastic syndromes, polycythemia veras, and non-melanoma skin cancers were excluded as second cancers. A cancer occurring after a non-melanoma skin cancer that followed the BC was classed as the second cancer rather than the non-melanoma skin cancer
Schottenfeld [15]	Treatment (rather than dx) of breast, endometrial, ovarian, vagina, vulva, or cervix uteri cancers at Memorial Sloan Kettering Cancer Centre between 1949 and 1962	Start: Unstated End: Unstated. Study ended in 1962	Retrospective cohort	The USA (Memorial Sloan Kettering Cancer Centre)	Patients with cancer of the breast, endometrium, ovary, vagina, vulva, or cervix uteri treated at the Memorial Sloan Kettering Cancer Centre between 1949 and 1962. Cohort was stratified by first cancer site, allowing analysis for first BC	The study predates the publication of the SEER SPC coding rules, the most common rules applied in North America. However, medical records were reviewed to validate the pathologic findings (where presumably recurrences and metastases were ruled out) whenever SPC incidence "increased significantly"
Silverman [24]	1990–2006	Start: At BC dx. Also provided results for a start of follow-up at 6 m after BC dx End: at SPC dx, death, or end of study (2011)	Retrospective cohort	Israel—Israel National Cancer Registry	Women with first BC, excluding breast lymphomas	SPC coding rules unstated, but Israel National Cancer Registry uses IARC/IACR rules with the following optional rules: 1: Two tumours of different laterality, but of the same morphology, diagnosed in paired organs (e.g. breast) are registered separately unless stated to have originated from a single primary 2: Cancers that occur in any 4th character subcategory of colon (C18) and skin (C44) are registered as multiple primary cancers
Tabuchi [25]	Dx 1985–2004	Start: 3 m after BC dx End: At SPC dx, death, 10y after BC dx, 80th birthday, or study end (2005)	Retrospective cohort	Japan (Osaka Cancer Registry)	All individuals aged 0–79 dx with a first primary cancer who survived at least 3 m. Cohort was stratified by first cancer site, allowing analysis for first BC	According to IARC/IACR rules. Only discordant second cancers included
Trama [16]	Dx at and followed up until various periods starting from 1976, respectively, according to the establishment dates of and the most recent incidence data entry dates of the registries in study	Start: At BC dx End: At SPC dx, death, emigration, or end of last year of data entry into registry records (dates varied by registry)	Retrospective cohort	Italy—34 cancer registries covering 43% of Italian population as of 2019	Individuals diagnosed with a first primary cancer (invasive or of uncertain behaviour), aged 15–39 at the first cancer diagnosis, who survived at least 5y after the first diagnosis. Cohort was stratified by first cancer site, allowing analysis for first BC	IARC/IACR rules
Tsukuma [26]	1966–86, but information on standardized incidence ratios for SPCs following BC only available for those dx 1978–86	Start: At BC dx End: At SPC dx, death, 80th birthday, or study end (1989)	Retrospective cohort	Japan (Osaka Cancer Registry)	All individuals aged 0–79 dx with a first primary cancer, who survived at least 3 m after the first cancer dx. Cohort was stratified by first cancer site, allowing analysis for first BC	IARC/IACR rules. Second cancers only included if they occurred at least 3 m after the BC dx
Utada [27]	1985–2007	Start: At BC dx End: At SPC dx, death, or study end (2008)	Retrospective cohort	Japan (Nagasaki Cancer Registry)	All individuals dx with a first primary cancer. Cohort was stratified by first cancer site, allowing analysis for first BC	IARC/IACR rules. Only discordant second cancers included
Zheng [17]	1958–2015	Start: At BC dx End: At SPC dx, death, emigration, or study end (2015)	Retrospective cohort	Sweden (FCD^10^)	The Swedish FCD is composed of two separate cohorts. 1: Swedish people born after 1931 (“offspring generation”), and 2: their parents (“parental generation”). This study examined the subset of the offspring generation dx with BC between 1958 and 2015	Swedish FCD data is linked to national registry, which uses IARC/IACR rules. All second cancers undergo "rigorous histological diagnostics". A request for separate and consistent tumour notifications from clinicians and pathologists is required

^1^Breast Cancer

^2^Diagnosis/diagnoses/diagnosed

^3^Follow-up

^4^International Association of Cancer Registries/International Agency for Research on Cancer

^5^Year/years

^6^Second Primary Cancer

^7^Surveillance, Epidemiology, and End Results

^8^Month/months

^9^European Prospective Investigation into Cancer and nutrition

^10^Family Cancer Database

**Table 2:** Further study characteristics

Author and publication year	Total person years	FU^1^ time since BC^2^ dx^3^ strata	Age strata at BC dx	Specific SPC^4^s for which SIR^5^s reported	N^6^ first BC/N SPCs	SIR (95% CI^7^) for combined risk of non-breast SPCs
AIRTUM Working Group [1]	1,274,882	0–1 m^8^, 2–11 m, 12–59 m, 60–119 m, > = 120 m	0–19, 20–29, 30–39, 40–49, 50–69, > = 70	Oral cavity, Pharynx, Larynx, Oesophagus, Stomach, Colon, Rectum, Liver, Gallbladder, Pancreas, Lung, Skin melanoma, Mesothelioma, Kaposi sarcoma, Soft tissue, Bone, Corpus Uteri, Cervix Uteri, Ovary, Kidney and renal pelvis, Bladder and urinary tract, Brain and central nervous system, Thyroid, Hodgkin’s lymphoma, Non-Hodgkin’s lymphomas, Multiple myeloma, Leukaemias (Lymphoid leukaemia, Myeloid leukaemia, Other leukaemias), Other sites	215,809/10597	1.12 (1.10–1.14)
Andersson [2]	256,563	1–9 y^9^, 10–19 y, > = 20 y	< 50, 50–59, 60–69, 70–89	Lip, Tongue, Salivary glands, Mouth, Pharynx, Oesophagus, Stomach, Small intestine, Colon, Rectum, Liver, Gallbladder, Pancreas, Nose (sinuses), Larynx, Lung, Pleura, Cervix Uteri, Corpus Uteri, Uterus (other), Ovary (uterine adnexa), Other female genital organs, Kidney, Bladder (and other unspecified related sites), Melanoma of skin, Eye, Brain and nervous system, Thyroid, Bone, Soft tissues, Non-Hodgkin’s Lymphoma, Hodgkin’s disease, Multiple myeloma, Acute leukaemia, Other leukaemia	31,818/1993	1.04 (0.99–1.08)
Brenner [3]	43,642.25	Unreported	< 50, > = 50	Stomach, Colon, Rectum, Gallbladder and bile ducts, Pancreas, Corpus Uteri, Cervix Uteri, Ovaries, Urinary bladder, Kidneys, Lymphomas and leukaemias	9678/206	0.84 (0.73–0.96)
Brown [4]	2,990,587	1–9 y, 10–19 y, 20–29 y, > = 30 y	< 40, 40–49, 50–64, > 64	Salivary gland, Oesophagus, Lung, Pleura, Thyroid, Bone, Connective tissue, Uterine corpus, Lip, Tongue, Mouth, Pharynx, Stomach, Small intestine, Colon, Rectum/anus, Liver, Pancreas, Gallbladder, Nose/nasal cavity, Larynx, Cervix, Ovary, Kidney, Bladder, Malignant Melanoma, Eye, Brain and central nervous system	376,825/23158	1.15 (1.14–1.17)
Chen (Germany) [5]	Unreported	Unreported	Unreported	Unreported	234,863 (male and female combined)/3676	1.15 (1.13–1.17). 1.15 is the midpoint of the reported 95% CI—it was taken as an approximation for the SIR due to early rounding in the study
Diab [6]	Unreported	Unreported	< 50, > = 50	Oral cavity and pharynx, Digestive system, Colon, rectum, and anus, Pancreas, Peritoneum, omentum and mesentery, Respiratory system, Bones and joints, Soft tissue including heart, Skin excluding basal and squamous, Breast, Female genital system, Corpus and uterus (not otherwise specified), Ovary, Urinary system, Brain and other nervous system, Endocrine system, Lymphoma, Leukaemia	514,479/45509	1.03 (1.02–1.04)
Evans [7]	832,958.1	Unreported	< 50, > = 50	Tongue, Mouth, Oesophagus, Stomach, Colon, Rectum, Liver, Gallbladder, Pancreas, Larynx, Lung and bronchus, Bone, Connective tissue, Skin melanoma, Cervix Uteri, Corpus Uteri, Ovary, Bladder, Kidney, Brain and nervous system, Thyroid, Non-Hodgkin’s Lymphoma, Multiple myeloma, Lymphoid Leukaemia, Myeloid Leukaemia	145,677/4470	0.89 (0.86–0.92)
Gulhan [18]	16,377	Unreported	Unreported	Endometrial, Ovary, Cervical	6356/88	1.76 (1.43–2.17)
Harvey [8]	271,524	< 1 y, 1–4 y, 5–9 y, > = 10 y	< 45, 45–54, > = 55	Lip, Tongue, Salivary gland, Gum and other mouth, Pharynx, Oesophagus, Stomach, Colon, Rectum, Liver (biliary), Pancreas, Nasal cavities and sinuses, Larynx, Trachea, bronchus, and lung, Cervix uteri, Corpus uteri, Uterus (not otherwise specified), Ovary and fallopian tubes, Kidney and renal pelvis and ureter, Bladder and other urinary, Skin (melanoma), Eye, Brain and central nervous system, Thyroid gland, Bone, Connective tissue, Non-Hodgkin’s lymphoma, Hodgkin’s disease, Multiple myeloma, Leukaemias, Chronic lymphocytic leukaemia, Acute nonlymphocytic leukaemia	41,109/2057	1.15 (1.10–1.20)
Hung [19]	527,009	< 1 y, 1–4 y, > = 5 y	20–29, 30–39, 40–49, 50–59, 60–69, 70–79, > = 80	Head and neck, Oesophagus, Stomach, Colon and rectum and anus, Liver and biliary tract, Liver, Lung and mediastinum, Bone and soft tissue, Skin, Cervix, Uterus, Ovary, Bladder, Kidney, Thyroid, Hematologic malignancies, All others	100,915/3,080	1.50 (1.44–1.55)
Jégu [9]	351,434	Unreported	Unreported	Corpus Uteri	Unreported/2476	1.31 (1.26–1.36)
Jung [20]	13,433.5	Unreported	30–39, 40–49, 50–59, 60–69, > = 70	Oesophagus, Stomach, Colon and rectum, Anus, Liver, Gallbladder and common bile duct, Lung, Cervix, Endometrium, Ovary, Kidney, Bladder, Thyroid, Hodgkin’s lymphoma, Non-Hodgkin’s lymphoma, Acute myeloid leukaemia, Skin, Muscle	3344/93	1.56 (1.27–1.91)
Lee [21]	290,966	< = 5 y, 6–10 y, > 10 y	< 50, > = 50	Bone, Corpus uteri, Ovary, Non-melanoma skin, Thyroid, Head and neck, Nasopharynx and nasal cavity, Oesophagus, Stomach, Small intestine, Colon and rectum, Liver, Biliary system, Pancreas, Lung, Thymus, Sarcoma, Cervix uteri, Urinary bladder, Kidney and other urinary organs, Brain, Leukaemia or lymphoma, Others	53,783/1085	1.09 (1.03–1.16)
Levi [10]	61,834	< 5 y, > = 5 y	Unreported	Mouth and pharynx, Oesophagus, Stomach, Colorectum, Gallbladder, Pancreas, Lung, Soft tissue, Skin melanoma, Cervix Uteri, Corpus Uteri, Ovary, Other female genital organs, Bladder, Kidney, Thyroid, Non-Hodgkin’s lymphomas, Multiple myelomas, Leukaemias, Other and unknown sites	9729/443	1.14 (1.04–1.25)
Mellemkjaer [33]	3,784,660	< 1 y, 1–9 y, > = 10y	< = 45, 46–55, > = 56	Oral cavity and pharynx, Oesophagus, Stomach, Small intestine, Colorectal, Liver, Pancreas, Larynx, Lung, Bone, Soft tissue sarcoma (of thorax and upper lim inc. shoulder), Melanoma, Non-melanoma skin cancer, Corpus Uteri, Ovary, Bladder, Kidney, Brain and nervous system, Thyroid gland, Non-Hodgkin’s lymphoma, Leukaemia, Myeloid leukaemia	525,527/31399	1.25 (1.24–1.26)
Molina-Montes [11]	37,605	< 5 y, > = 5 y	< 50, > = 50	Endometrium, Colon and rectum, Stomach, Ovary, Thyroid gland, Non-melanoma skin, Kidney, Bladder, Hematologic malignancies (lymphoid leukaemia, myeloid leukaemia, and multiple myeloma)	5897/314	1.39 (1.24–1.55)
Murakami [22]	53,738	< 1 y, 1–4 y, 5–9 y, > = 10 y	< 45, 45–54, > = 55	Buccal cavity, Stomach, Oesophagus, Colon, Rectum, Liver, Pancreas, Lung, Cervix Uteri, Corpus Uteri, Ovary, Urinary bladder, Thyroid gland, Leukaemia	9503/254	1.34 (1.18–1.52)
Odani [23]	266,685	3 m–1 y, 1–5 y, 5–10 y	Unreported	Oral cavity/pharynx, Stomach, Colorectum, Liver, Gallbladder, Pancreas, Lung, Uterus, Ovary, Kidney/urinary tract/bladder, Thyroid, Blood	47,622/1843	1.84 (1.76–1.92)
Ricceri [12]	56,496	Unreported	Unreported	Colorectum, Lung, Pancreas, Melanoma, Endometrium, Ovary, Kidney, Thyroid gland, Lymphomas	10,045/352	1.30 (1.18–1.42)
Rubino [13]	33,044	1–10 y, > = 10 y	< 50, > = 50	Oral cavity, Oesophagus, Stomach, Colon and rectum, Liver and gallbladder, Pancreas, Larynx, Lung and bronchus, Uterus, Ovaries, Bladder, Kidney, Melanoma, Nervous system, Thyroid, Other endocrine, Bone, Soft tissue, Undefined sites, Myeloma, Lymphoma, Leukaemia	4416/193	1.40 (1.21–1.62)
Schaapveld [14]	362,470	Not reported in a fashion that allows accurate extraction of age-stratified SIRs and corresponding 95% standard errors	< 50, > = 50	Head and neck, Thyroid, Oesophagus, Stomach, Pancreas, Gallbladder/extrahepatic bile ducts, Colon, Rectum and Anus, Lung, Soft tissue sarcomas, Melanoma of skin, Ovary, Cervix, Uterus, Vulva, Kidney, Bladder, Brain, Acute myeloid leukaemia, Other Leukaemia, Non-Hodgkin’s lymphoma, Multiple myeloma	58,068/2578	1.22 (1.17–1.27)
Schottenfeld [15]	Unreported	Unreported	Unreported	Ovary, Corpus Uteri, Cervix Uteri, Vulva and vagina, Buccal cavity and Pharynx, Oesophagus, Stomach, Colon, Rectum, Pancreas, Liver and bile ducts, Larynx, Lung, Kidney, Bladder, Lymphoma and leukaemia, Salivary glands, Thyroid, Soft-part sarcomas, Bone sarcomas	9792/231	1.01 (0.9–1.1)
Silverman [24]	363,333	Unreported	< 50, > = 50	Colorectum, Uterus, Lung, Ovary, Non-Hodgkin's Lymphoma, Brain, Melanoma (invasive), Thyroid, Leukaemia, Uterine Cervix	43,794/3866	1.26 (1.23–1.30)
Tabuchi [25]	197,571	< 1 y, 1–5 y, 5–10 y	Unreported	Mouth/pharynx, Stomach, Oesophagus, Colorectal, Liver, Gallbladder, Pancreas, Lung, Uterus, Ovary, Thyroid, Kidney/urinary tract/bladder, Blood	Unreported/1007	1.48 (1.39–1.57)
Trama [16]	102,629	< 5 y, 5–10 y, 10–15 y, 15–20 y, 20–25 y, > 25 y	Unreported	Soft tissue sarcomas, Colorectal, Stomach, Pancreatic, Liver, Bladder, Kidney, Cervical, Ovarian, Corpus Uteri, Central nervous system, Germ cell	11,328/299	1.13 (1.0–1.3)
Tsukuma [26]	Unreported	< 1 y, 1–4 y, 5–9 y	Unreported	Stomach, Colon, Lung, Thyroid	Unreported/226	1.42 (1.25–1.62)
Utada [27]	Unreported	Not reported in a fashion that allows accurate extraction of age-stratified SIRs and corresponding 95% standard errors	Unreported	Lung, Uterus, Ovary, Thyroid	Unreported/727	1.16 (1.08–1.25)
Zheng [17]	Unreported	Unreported	Unreported	Upper aerodigestive tract, Oesophagus, Stomach, Small intestine, Colorectum, Anus, Liver, Nose, Pancreas, Lung, Cervix, Endometrium, Uterus, Ovary, Other female genitals, Kidney, Bladder, Melanoma, Skin (squamous cell carcinoma), Eye, Nervous system, Thyroid gland, Endocrine gland, Bone, Connective Tissue, Non-Hodgkin’s lymphoma, Hodgkin’s lymphoma, Myeloma, Leukaemia, Cancer of unknown primary, Colon, Rectum	87,752/6299	1.43 (1.40–1.47)

^1^Follow-up

^2^Diagnosis/Diagnoses/Diagnosed

^3^Breast Cancer

^4^Second Primary Cancer

^5^Standardized Incidence Ratio

^6^Number (of)

^7^Confidence Interval

^8^Month/Months

^9^Year/Years

## Results of meta-analyses

### Unstratified results

The unstratified meta-analysis consisted of nineteen studies [1, 3, 5–7, 9–11, 13–15, 18–20, 23, 24, 26, 27, 33]. All but two [3, 7] reported an increase in SPC risks following a first primary BC.

**Fig. 2:**
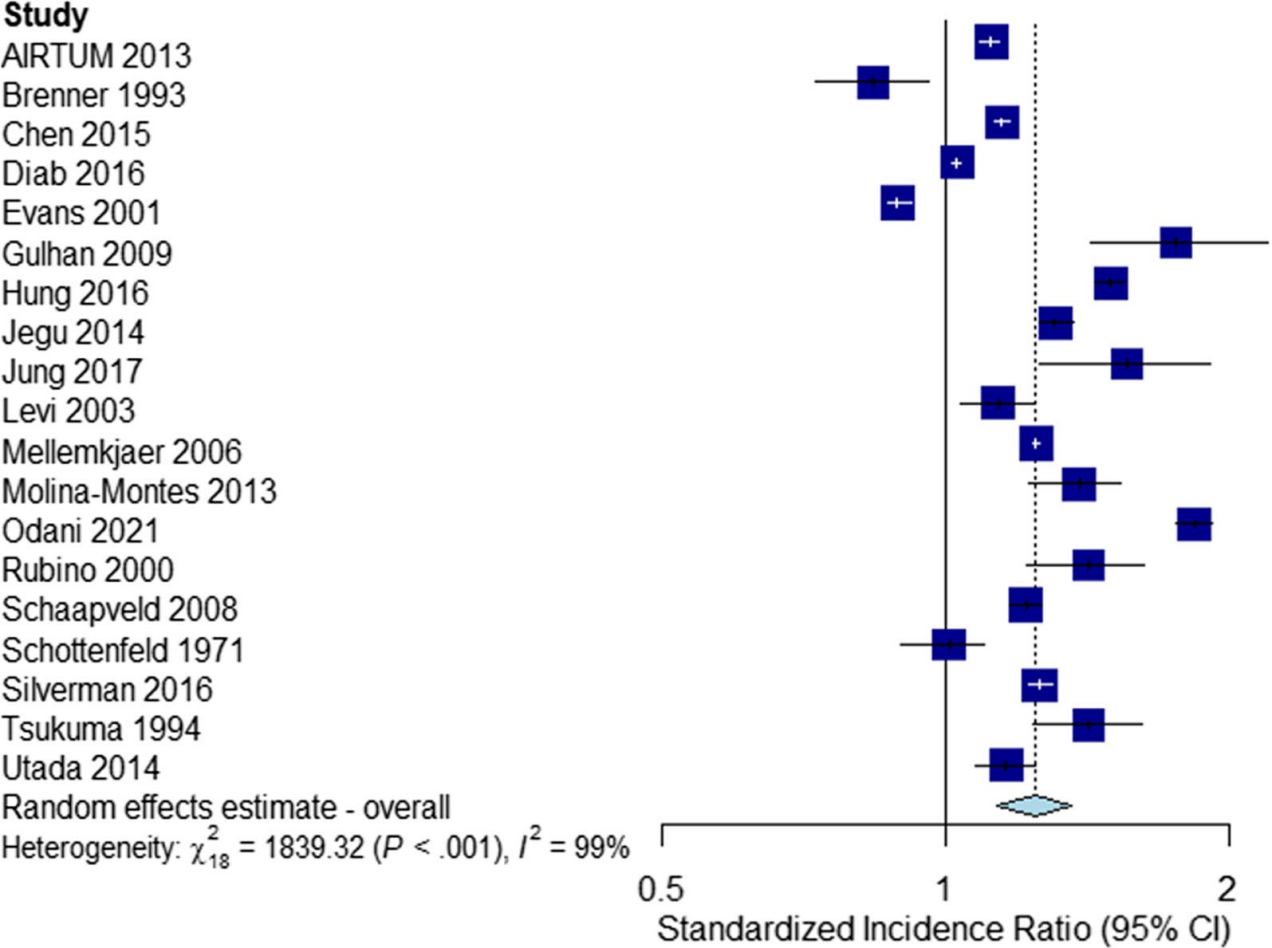
Second non-breast primary risks following first primary breast cancers

The summary SIR was estimated as 1.24 (95% CI 1.14–1.36, Fig. 2). Significant evidence for heterogeneity was found (*Q*: 1839.32, *I*^2^: 99%, *p* < 0.001).

Following leave-one-out analyses, we found the studies by Diab et al. [6], Odani et al. [23], Mellemkjær et al. [33], Evans et al. [7], and Hung et al. [19] to contribute the most to heterogeneity, with Cochran’s *Q* falling by 40%, 23%, 20%, 15%, and 13% in the meta-analyses consisting of all studies in the unstratified meta-analysis other than the respective study under investigation. Eliminating all these studies did not appreciably affect the summary SIR estimate (SIR: 1.24, 95% CI 1.13–1.35), and there remained significant evidence for heterogeneity (*Q*: 154.89, *I*^2^: 92%, *p* < 0.001).

We identified 7 outlier studies [3, 6, 7, 15, 18, 19, 23]. Eliminating all outlier studies also had little effect on the SIR estimate (SIR: 1.25, 95% CI 1.19–1.31), and significant evidence for heterogeneity was still present (*Q*: 166.23, *I*^2^: 93%, *p* < 0.001).

Examining a funnel plot and performing Egger’s test revealed no significant evidence of publication bias (Additional file 1).

### Effects of geographic region

**Fig. 3:**
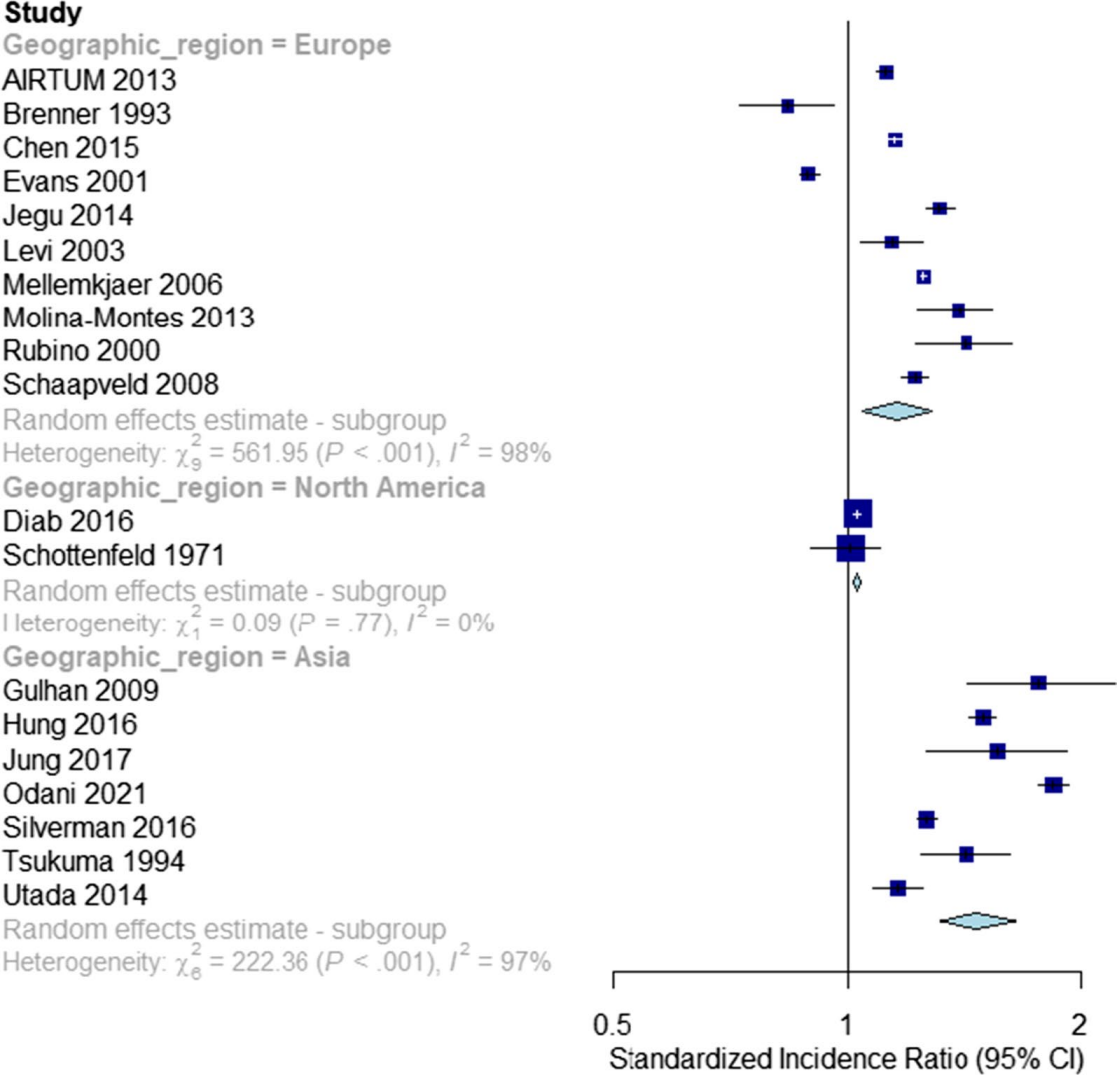
Second non-breast primary risks following first primary breast cancers. Stratification: geographic region

We found significant evidence that summary SIRs varied by geographic region (SIR: 1.47, 95% CI 1.29–1.67 for Asian studies vs. 1.16 (1.04–1.28) for European studies vs. 1.03 (1.02–1.04) for North American studies, *p* for difference: < 0.001, Fig. 3).

Significant heterogeneity was found for the Asian subgroup analysis (*Q*: 222.36, *I*^2^: 97%, *p* < 0.001) and for the European subgroup analysis (*Q*: 561.95, *I*^2^: 98%, *p* < 0.001). No significant evidence for heterogeneity was found in the North American subgroup analysis (*Q*: 0.09, *I*^2^: 0%, *p*: 0.77).

There was significant evidence that Asian BC survivors had higher SPC risks in comparison with European BC survivors, for whom the largest amount of data was available (*p* for difference: 0.005). There was also significant evidence that American BC survivors were at lower risks of SPCs compared to European BC survivors (*p* for difference: 0.027).

### Effects of age at BC onset

**Fig. 4:**
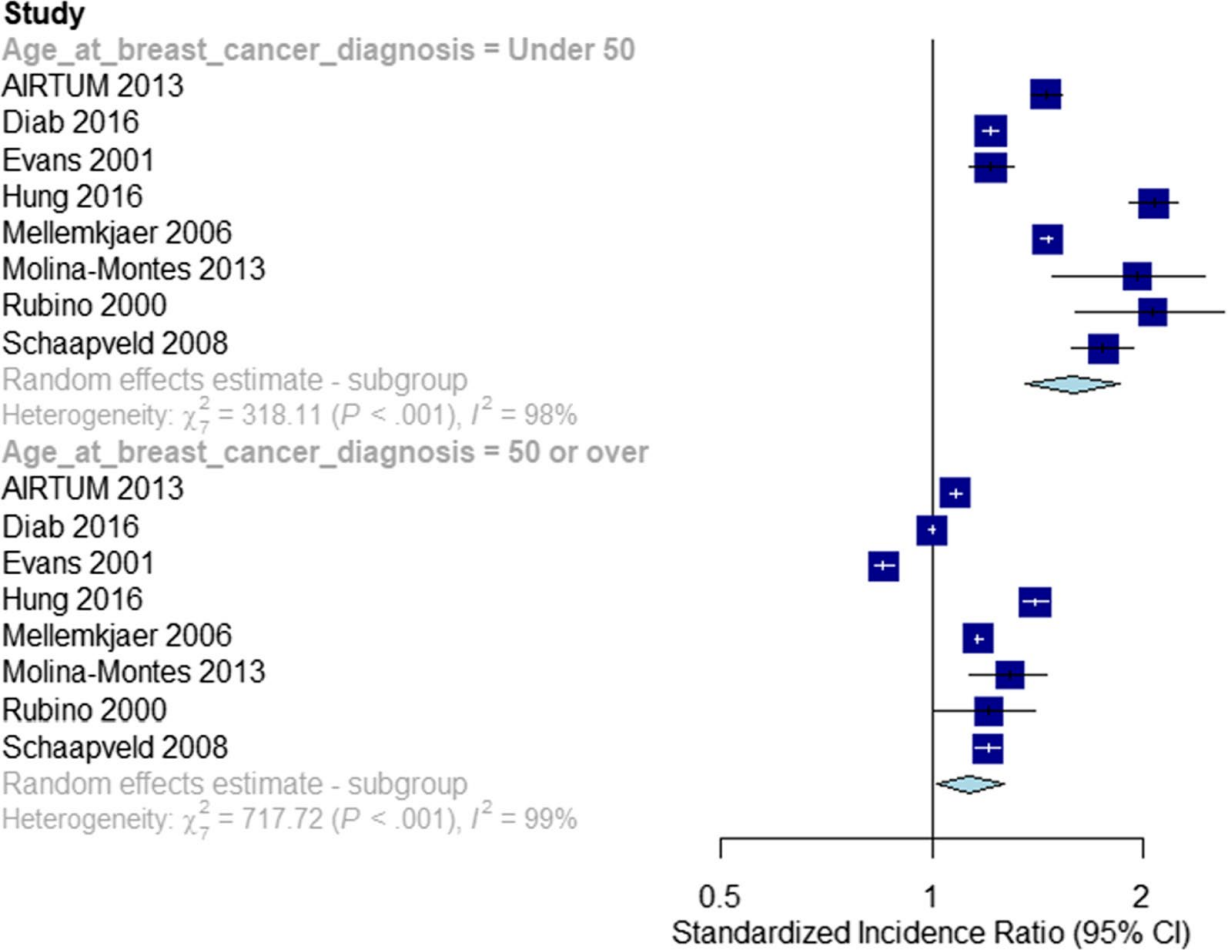
Second non-breast primary risks following first primary breast cancers. Stratification: age at breast cancer diagnosis

Eight studies were included in the age-stratified meta-analyses [1, 6, 7, 11, 13, 14, 19, 33]. One small study also stratified by age at breast cancer diagnosis but was not included in this analysis due to a discrepancy between the number of SPCs reported in total and within each age stratum [20]. SPC risks were significantly elevated in both age groups compared to the risks of first primaries, and there was significant evidence for a difference in summary SIRs between these groups (SIR: 1.59, 95% CI 1.36–1.85 for those aged under 50 at first BC diagnosis vs. 1.13 (95% CI 1.01–1.26) for those aged over 50 at first BC diagnosis, *p* for difference: < 0.001, Fig. 4). Heterogeneity was present in both strata (Aged under 50 at first BC diagnosis: *Q*: 318.11, *I*^2^: 98%, *p* < 0.001. Aged 50 or over at first BC diagnosis: *Q*: 717.72, *I*^2^: 99%, *p* < 0.001).

### Effects of follow-up time duration

Stratification of BC survivors by follow-up duration revealed no significant evidence for a difference in SPC risks. Full results may be seen in the Additional file 1.

### Second primary risks at specific sites

Point estimates of summary SIRs estimating SPC risks unstratified by age at the nineteen examined sites ranged from 0.80 (for the brain and CNS) to 1.89 (for the thyroid). BC survivors were found to be at significantly lower risk of brain and CNS cancers (SIR: 0.80, 95% CI 0.71–0.91), and there was a suggestion of decreased cervix uteri cancer risk (SIR: 0.88, 95% CI 0.77–1.00). In contrast, there was significant evidence for elevated second primary bladder (SIR: 1.15, 95% CI 1.05–1.26), corpus uteri (SIR: 1.84, 95% CI 1.53–2.23), kidney (SIR: 1.43, 95% CI 1.17–1.73), blood (leukaemia) (SIR: 1.30, 95% CI 1.17–1.45), lung (SIR: 1.25, 95% CI 1.03–1.51), skin (melanoma) (SIR: 1.34, 95% CI 1.18–1.52), oesophagus (SIR: 1.39, 95% CI 1.26–1.55), ovary (SIR: 1.53, 95% CI 1.35–1.73), stomach (SIR: 1.23, 95% CI 1.12–1.36), and thyroid (SIR: 1.89, 95% CI 1.49–2.38) cancer risks following BC.

We found BC survivors first diagnosed with BC at under age 50 to be at elevated risk of second primaries at the bladder (SIR: 1.32, 95% CI 1.17–1.48), blood (leukaemia) (SIR: 1.91, 95% CI 1.77–2.05), corpus uteri (SIR: 1.40, 95% CI 1.12–1.76), kidney (SIR: 1.29, 95% CI 1.15–1.43), lung (SIR: 1.65, 95% CI 1.49–1.82), oesophagus (SIR: 2.21, 95% CI 1.89–2.60), ovary (SIR: 2.24, 95% CI 1.59–3.13), pancreas (SIR: 1.35, 95% CI 1.16–1.57), skin (melanoma) (SIR: 1.34, 95% CI 1.23–1.45), stomach (SIR: 1.90, 95% CI 1.75–2.06), and thyroid (SIR: 2.06, 95% CI 1.83–2.31).

We found there to be significantly increased risks of second primaries at three sites in BC survivors diagnosed with BC at age 50 or over: the corpus uteri (SIR: 1.75, 95% CI 1.29–2.37), the oesophagus (SIR: 1.20, 95% CI 1.06–1.37), and the skin (melanoma) (SIR: 1.25, 95% CI 1.17–1.35).

BC survivors diagnosed with breast cancer before age 50 were at significantly increased risk of second primary lung cancer compared to BC survivors diagnosed with breast cancer at age 50 or over (SIR: 1.65, 95% CI 1.49–1.82 for those aged under 50 at first BC diagnosis vs. 0.81 (95% CI 0.55–1.20) for those aged over 50 at first BC diagnosis, *p* for difference: < 0.001). They were also at significantly increased risks of second primaries at the pancreas (SIR: 1.35, 95% CI 1.16–1.57 vs. 0.92 (95% CI 0.81–1.04), *p* for difference: < 0.001), blood (leukaemia) (SIR: 1.91, 95% CI 1.77–2.05 vs. 1.34 (95% CI 0.99–1.81), *p* for difference: 0.026), oesophagus (SIR: 2.21, 95% CI 1.89–2.60 vs. 1.20 (95% CI 1.06–1.37), *p* for difference: < 0.001), ovary (SIR: 2.24, 95% CI 1.59–3.13 vs. 1.04 (95% CI 0.93–1.16), *p* for difference < 0.001), stomach (SIR: 1.90, 95% CI 1.75–2.06 vs. 1.10 (95% CI 0.91–1.34), *p* for difference < 0.001), and thyroid (SIR: 2.06, 95% CI 1.83–2.31 vs. 1.17 (95% CI 0.90–1.52), *p* for difference < 0.001).

Full results may be seen in Table 3.

**Table 3:** Risks of second primaries at specific sites

Cancer site	SIR (95% CI)—breast cancer diagnosed at any age	SIR (95% CI)—breast cancer diagnosed at under age 50	SIR (95% CI)—breast cancer diagnosed at age 50 or over	Number of studies in meta-analysis		
				Unstratified by age at BC dx	Aged under 50 at BC dx	Aged 50 or over at BC dx
Bladder^1^	1.15 (1.05–1.26)	1.32 (1.17–1.48)	1.08 (0.89–1.30)	8	4	4
Blood (leukaemia)^2^	1.30 (1.17–1.45)	1.91 (1.77–2.05)	1.34 (0.99–1.81)	8	4	4
Blood (myeloma)^3^	0.83 (0.68–1.02)	1.01 (0.53–1.94)	0.63 (0.48–0.82)	4	1	1
Blood (non-Hodgkin’s lymphoma)	1.04 (0.91–1.19)	1.17 (0.96–1.42)	0.93 (0.65–1.33)	7	2	2
Brain and central nervous system^4^	0.80 (0.71–0.91)	0.95 (0.81–1.11)	0.75 (0.69–0.81)	7	4	3
Cervix uteri^5^	0.88 (0.77–1.00)	0.65 (0.46–0.93)	0.57 (0.23–1.39)	10	2	2
Colorectum^6^	1.12 (0.99–1.27)	1.30 (0.91–1.86)	1.02 (0.87–1.19)	11	5	5
Corpus uteri^7^	1.84 (1.53–2.23)	1.40 (1.12–1.76)	1.75 (1.29–2.37)	16	5	5
Gallbladder^8^	1.13 (0.68–1.87)	0.49 (0.12–1.96)	0.86 (0.63–1.17)	7	1	1
Kidney^9^	1.43 (1.17–1.73)	1.29 (1.15–1.43)	1.35 (0.95–1.92)	11	4	4
Liver^10^	0.86 (0.60–1.24)	0.93 (0.71–1.21)	0.56 (0.33–0.96)	7	1	2
Lung^11^	1.25 (1.03–1.51)	1.65 (1.49–1.82)	0.81 (0.55–1.20)	12	3	3
Oesophagus	1.39 (1.26–1.55)	2.21 (1.89–2.60)	1.20 (1.06–1.37)	9	3	3
Ovary	1.53 (1.35–1.73)	2.24 (1.59–3.13)	1.04 (0.93–1.16)	16	6	6
Pancreas	1.09 (0.93–1.27)	1.35 (1.16–1.57)	0.92 (0.81–1.04)	11	3	4
Skin (melanoma)	1.34 (1.18–1.52)	1.34 (1.23–1.45)	1.25 (1.17–1.35)	7	3	3
Stomach	1.23 (1.12–1.36)	1.90 (1.75–2.06)	1.10 (0.91–1.34)	13	4	4
Thyroid	1.89 (1.49–2.38)	2.06 (1.83–2.31)	1.17 (0.90–1.52)	14	4	3
Vulva^12^	0.92 (0.63–1.35)	–	–	2	0	0

^1^Meta-analysis also includes data on cancer risks at the “urinary bladder”

^2^Meta-analysis includes data on combined lymphoid leukaemia and myeloid leukaemia risks

^3^Meta-analysis only includes data on “multiple myeloma(s)” risks

^4^Meta-analysis also includes data on cancer risks at the “brain and nervous system”, brain only, and nervous system only

^5^Meta-analysis also includes data on “cervical”, “cervix”, and “uterine cervix” cancer risks

^6^Meta-analysis includes data on combined colon and rectum cancer risks

^7^Meta-analysis also includes data on cancer risks at the “uterus” and “endometrium”

^8^Meta-analysis also includes data on cancer risks at the “gallbladder and bile ducts”, “gallbladder and common bile duct”, and “gallbladder/extrahepatic bile ducts”

^9^Meta-analysis also includes data on cancer risks at the “kidney and renal pelvis”

^10^Meta-analysis also includes data at the “liver and biliary tract” and “liver and bile ducts”

^11^Meta-analysis also includes data at the “lung and bronchus”

^12^Meta-analysis also includes data at the “vulva and vagina”

## Discussion

In this review, we found significant evidence for elevated SPC risks among BC survivors, particularly when first diagnosed with BC at under age 50 or in Asian hospitals/registries. Risks of second primary bladder, kidney, blood, lung, skin (melanoma), oesophagus, ovary, stomach, thyroid, and corpus uteri cancers were significantly increased, whereas risks of brain and CNS and cervix uteri SPCs were significantly decreased.

This review has several strengths. The studies were of high quality (Additional file 1), and we found no significant evidence for publication bias (Additional file 1). It includes an array of studies with large sample sizes [1, 4–7, 14, 17, 19, 21, 33], long follow-up periods [1, 2, 4, 6–8, 10, 13, 16, 17, 20, 21, 25, 27, 33], and recently updated data [1, 5, 6, 16, 17, 19, 20, 23]. Another strength is the inclusion of several studies from outside Europe and North America [18–27], allowing comparisons between regions with different demographics and BC incidence rates [59].

There are two main weaknesses of this review. The first is the high level of heterogeneity observed, and the second is the underreporting of potentially confounding risk factors.

Regarding the first point, much of the heterogeneity was contributed by Diab et al. [6], a very large study from North America, and the only study that was explicitly stated to use the SEER multiple tumour coding rules. It is therefore possible that the differences between such rules could account for some of the between-study differences in SPC risks, such as the significantly decreased SPC risks among North American studies compared to European studies. This would be at odds with the small study by Coyte et al. [42], which found non-breast SPC counts to be close to identical under both the SEER and the IARC/IACR rules. Larger studies comparing SPC counts observed under these two common sets of guidelines would help clarify this issue. Any differences in the ratio of the screening intensity for non-breast second primaries among BC survivors and the screening intensity for non-breast first cancers, or in the rates of risk-reducing surgeries performed in BC survivors, between North American and European populations could also partly explain these differences in SPC risks. However, this information was not reported in the studies. However, even if such discrepancies do account for the majority of the heterogeneity contributed by Diab et al., this would not explain the rest of the heterogeneity, which remained significant even following the elimination of four further studies identified as major drivers of heterogeneity [7, 19, 23, 33].

To investigate whether the definition of SPC influences the results, we also performed a meta-analyses including only studies using IACR/IARC coding rules to identify second primaries [1, 2, 5, 9–11, 14, 18, 23, 24, 26, 27, 33]. The summary SIR estimate was similar to the meta-analysis including all studies (All studies: SIR = 1.24, 95%CI = 1.14–1.36 vs. IARC/IACR studies: SIR = 1.27, 95%CI = 1.14–1.41), and there remained significant evidence of heterogeneity (All studies: Cochran’s *Q*: 1839.32, *I*^2^: 99%, *p* value: < 0.0001 vs. IARC/IACR studies: Cochran’s *Q*: 507.29, *I*^2^: 98%, *p* value: < 0.0001).

It is likely that including studies from three different continents contributed to heterogeneity, since SPC risks in these continents were found to vary significantly. Similarly, if ages at BC diagnoses varied widely between studies, then this would account for some of the heterogeneity, as younger age groups were found to be at significantly increased risk in comparison with those older. However, although heterogeneity was attenuated, it remained significant among Asian and European studies as well as in both younger age and older age groups, so these points cannot fully explain the observed heterogeneity.

It is also possible that differences in the treatments administered between studies could affect SPC risks [60–62] and thus contribute to heterogeneity. Unfortunately, this could not be assessed in this review since treatment effects were generally unreported. Information on other important variables also tended to be unavailable. For example, there was a paucity of information reported on obesity, tobacco intake, alcohol intake, the pathology of the initial BC, or family history of BC, which are known to influence cancer risks. We cannot therefore rule out confounding in the results due to these unreported confounding variables, nor can we rule out that unreported risk factors contributed to the significant heterogeneity observed.

It is known that cancer survivors may be more prone to being diagnosed with second cancers simply due to increased surveillance for cancer development, rather than a genuine increase in risk compared to the general population. This is known as “detection bias”, and we cannot rule out that it may have affected some results in this review [1]. However, many studies were included that excluded SPCs diagnosed within some time period following the first BC [2–4, 8–11, 13, 18, 20–23, 25, 26] when detection bias is likely to be most pronounced [1]. Therefore, detection bias is unlikely to be a major weakness of this review.

It is also possible that some of the observed variability in SIRs between studies could be due to differences in analytical methods and differences in the data quality control processes or the definition of second primary cancers used across registries. For example, Diab et al. calculated SIRs using the SEER database, a population-based data set of very high quality [6, 63] and with a very limited amount of missing data [64]. Several large studies also drew their data from large European registries of similar standard [1, 5, 9, 14, 33]. All studies in the meta-analyses which reported the specific data source used to calculate the SIRs used population-based registry data, which in principle would be of similar good quality [1, 10, 11, 14, 15, 19, 20, 24, 27, 33]. However, most did not report on the exact quality control processes applied and the data missingness. Furthermore, a large study included in the meta-analyses included second and subsequent primaries in the calculations of reference incidences used to generate expected cancer counts [1], whereas others included only first cancers [5, 9], although this information was generally not reported. Excluding these estimates did not have a marked effect on SIR estimates [1].

Finally, although every effort was made to capture all relevant studies, it cannot be ruled out that some studies were not found or were excluded erroneously.

This review adds to the previously published review [34] in several ways. Firstly, the previous review included no studies published since June 2013, whereas this updated review included twelve studies published since [1, 5, 6, 9, 12, 16, 17, 19, 20, 23, 24, 27]. This review also includes studies with cohorts consisting of survivors of any given set of initial cancers provided SPC risks could be extracted for the subset of BC survivors, yielding three new studies published before June 2013 [15, 25, 26]. In total, eighteen of the twenty-eight studies in this review were not included in the previous review [1, 4–6, 8, 9, 12, 15–20, 23–27], including several large multicentre studies and two sizeable monographs [1, 4–6, 8, 9, 12, 16]. Several of the new studies are drawn from Asian registries [18–20, 23, 24, 27] and North American registries [6, 8, 15], whereas the previous review did not include any North American studies. This enabled us to assess differences in SPC risks between these geographic regions. Finally, the previous review found follow-up duration to significantly affect SPC risks, whereas this updated review found no significant evidence of this (Additional file 1). The overall summary female SIR of 1.24 (95% CI 1.14–1.36) is slightly higher than the summary SIR reported in the previous review (1.17 (95% CI 1.10–1.25)).

The increased SPC risks could be partly due to treatment effects of the initial BC, such as the administration of hormonal therapy such as tamoxifen, or the administration of chemotherapy or radiotherapy [60–62, 65]. The latter may explain the increased risks of second oesophagus and lung primaries in BC survivors diagnosed at under age 50, as radiotherapy confers increasing risks of lung and oesophagus primaries with time since administration [63]. Similarly, chemotherapy is associated with increased leukaemia risk [66, 67] and is more commonly administered to younger BC survivors [68], possibly explaining the significantly higher risks of second primary leukaemias we found for this group. Shared risk factors between breast and other cancers such as obesity will also contribute to the elevated SPC risks among BC survivors [69, 70]. For example, thyroid cancer risks may be elevated by obesity or hormonal risk factors shared with BC [38]. The increased risk of SPCs at the lung [71], in the urogenital system [71] in the gastrointestinal system [71], and at other sites [12, 71, 72] may potentially be associated with increased smoking among BC survivors in comparison with the general population [73].

Germline susceptibility to BC may also raise specific SPC risks [74]. For example, pathogenic variants in known BC susceptibility genes are associated with risks for other cancers. Pathogenic variants in *BRCA1/2* have been found to be associated with risks of multiple primary cancers, including pancreatic and stomach cancers [75]. Pathogenic variants in *BRCA1/2* are also associated with ovarian cancer risk [76, 77]*,* as are pathogenic variants in *PALB2* [78], *RAD51C* [79, 80], and *RAD51D* [80, 81]. Such observations may explain the elevated ovarian SPC risks found in this review, particularly among younger BC survivors [82, 83]. There also exist common genetic variants with pleiotropic effects, associated with elevated breast and ovarian cancer risks [84]. Elevated polygenic risk scores are often associated with risks for more than one cancer [84]; for example, a BC polygenic risk score has been associated with colorectal cancer risk [85] and a recent large study found the prevalence of pathogenic protein-truncating variants in established BC susceptibility genes among female BC survivors to be 5.6% [86]. Genetic susceptibility could therefore account for a notable proportion of second primaries following BC in women.

If germline susceptibility does increase SPC risk in female BC survivors, this may partly explain our finding of elevated SPC risks in women diagnosed with BC at under age 50 compared to those diagnosed when older, since genetic susceptibility to BC is associated with earlier BC diagnosis [82, 87]. This finding will also partly account for the increased SPC risks among those diagnosed with BC in Asian registries, as BC is generally diagnosed at younger ages in Asia [88, 89].

The decreased risks of blood (myeloma), brain and CNS, and liver SPCs among BC survivors aged 50 or over at first BC diagnosis may be explained by under-ascertainment of SPCs in older age groups [7]. We also found brain and CNS SPC risks to be significantly decreased when unstratified by age, which may be attributable to misclassifications of second primaries as metastases [90].

## Conclusions

In conclusion, this review found that the combined risks of second non-breast cancer following a first primary BC were significantly elevated. Female BC survivors aged under 50 at BC onset or who were from Asian registries/hospitals were found to be at higher risks than other groups. Finally, we found second cancers at the bladder, corpus uteri, kidney, blood, lung, skin (melanoma), oesophagus, ovary, stomach, and thyroid to notably contribute to the observed elevated SPC risks.

The results may lead to increased awareness of the magnitudes and distribution by site of SPC risks following BC. They could also better inform cancer risk management, although specific recommendations would be beyond the scope of this review.

## Supplementary Information

**Additional file 1.** Additional File.

## Data Availability

All data generated or analysed during this study are included in the previously referenced published articles [1–27, 33] (and their supplementary information files).

## Abbreviations

SPC: Second primary cancer
BC: Breast cancer
SIR: Standardized incidence ratio
SEER: Surveillance, Epidemiology and End Results
IACR: International Association of Cancer Registries
IARC: International Agency for Research on Cancer
OMCC: Osaka Medical Centre for Cancer and Cardiovascular Diseases
OCR: Osaka Cancer Registry
CI: Confidence interval
CNS: Central nervous system
NOS: Newcastle–Ottawa Scale
BRCA1/2: BReast CAncer gene 1/2
PALB2: Partner and localizer of BRCA2
RAD51C: RAD51 paralog C
RAD51D: RAD51 paralog D
