# Supplementary material for: Risks of second non-breast primaries following breast cancer in women: a systematic review and meta-analysis
Source: Breast Cancer Res. 2023 Feb 10;25:18. doi: 10.1186/s13058-023-01610-x (PMC9912682; doi:10.1186/s13058-023-01610-x)
Supplement: Supplementary file 1 — Additional file 1. Additional File. [file 13058_2023_1610_MOESM1_ESM.docx]

Additional File

Contained within this file are an evaluation of publication bias, a delineation of the methods used to assess study quality and the corresponding results, and full results of the meta-analyses stratified by follow-up duration.

# Evaluation of publication bias

As can be seen in figure A1, no clear indication of publication bias was visible after plotting standard errors against SIRs. Accordingly, we found no significant evidence of publication bias after performing Egger’s test (p = 0.19).

# Evaluation of study quality

A study can receive a maximum score of nine on the Newcastle-Ottawa scale (NOS) (54). A score closer to the maximum is intended to indicate higher methodological quality. To be assigned the maximum score, a study must be assigned a score of four for the “selection” of their cohort of BC survivors, a score of two for the “comparability” of their cohort to the general population, and a score of three for their ascertainment of the “outcome” of interest (SPC development).

## Methods

For a study to be assigned a score of four for “selection”, their cohort of BC survivors must be representative of the average BC survivor in the relevant community, where the “community” refers to the population to which the study compared the BC survivors. In addition, the expected SPC counts had to be calculated using the same data source as the observed SPC counts, reliable sources had to be used to establish the BC diagnoses, and primaries prior to the BC had to have been eliminated. To be assigned a score of two for “comparability”, a study had to adjust for the calendar period and age group when estimating expected cancer counts. Finally, for a study to be assigned the maximum score for “outcome”, it had to establish any SPC diagnoses from record linkages to the initial BC diagnosis records, have a follow-up duration of 20 years or more, and have lost no more than 5% of the initial cohort to follow-up.

## Results

The breakdown of assigned NOS scores are included in Table A1.

Fourteen studies lost a point for selection due to estimating expected cancer counts from slightly different data sources than those which the observed counts were taken from, or due to leaving this information unstated (2,9,10,11,13–16,18–21,25,26). Four studies lost a point (4,6,13,20) due to a lack of clarity about their practices regarding primaries diagnosed prior to the first BC. One study (2) lost a point due to only including breast cancer survivors who met eligibility criteria for treatment under specific protocols, since this was deemed to render the cohort insufficiently representative of general age-matched female breast cancer survivors.

Three studies (12,17,20) lost a point for comparability due to not stating any measure to control for age when estimating expected primary counts. Eight studies (6,11,17,20,23–26) lost a point due to not stating any measure to control for calendar period.

All but one study (15) lost a point for outcome category due to reporting a loss of over 5% of their cohort to follow-up (13), or due to leaving this information unstated. Eleven studies lost a point because their follow-up duration was under 20 years (3,5,9,12–15,18,19,22,23).

# Effects of follow-up time duration: full results

We found no significant evidence that the risks of non-breast SPCs differed in the first 5 years following a BC diagnosis compared to later years (SIR: 1.57, 95%CI: 1.11-2.21 in the first 5 years vs 1.29 (1.01-1.66) thereafter, p for difference: 0.38, Figure A2). We observed significant heterogeneity in both strata (First 5 years: Q: 485.27, I^2^: 99%, p < 0.001. Thereafter: Q: 272.35, I^2^: 98%, p < 0.001).

Similarly, no significant evidence was found for a difference between SPC risks within the first 10 years following a BC diagnosis and SPC risks beyond this period (SIR: 1.35, 95%CI: 1.12-1.62 in the first 10 years vs 1.35 (1.18-1.55) thereafter, p for difference: 0.99, Figure A3). We also found heterogeneity in each of these strata (First 10 years: Q: 417.77, I^2^: 99%, p < 0.001. Thereafter: Q: 43.25, I^2^: 95%, p < 0.001).

# Notes

*It should be noted that all abbreviations and reference numbers correspond to those used in the primary manuscript.*


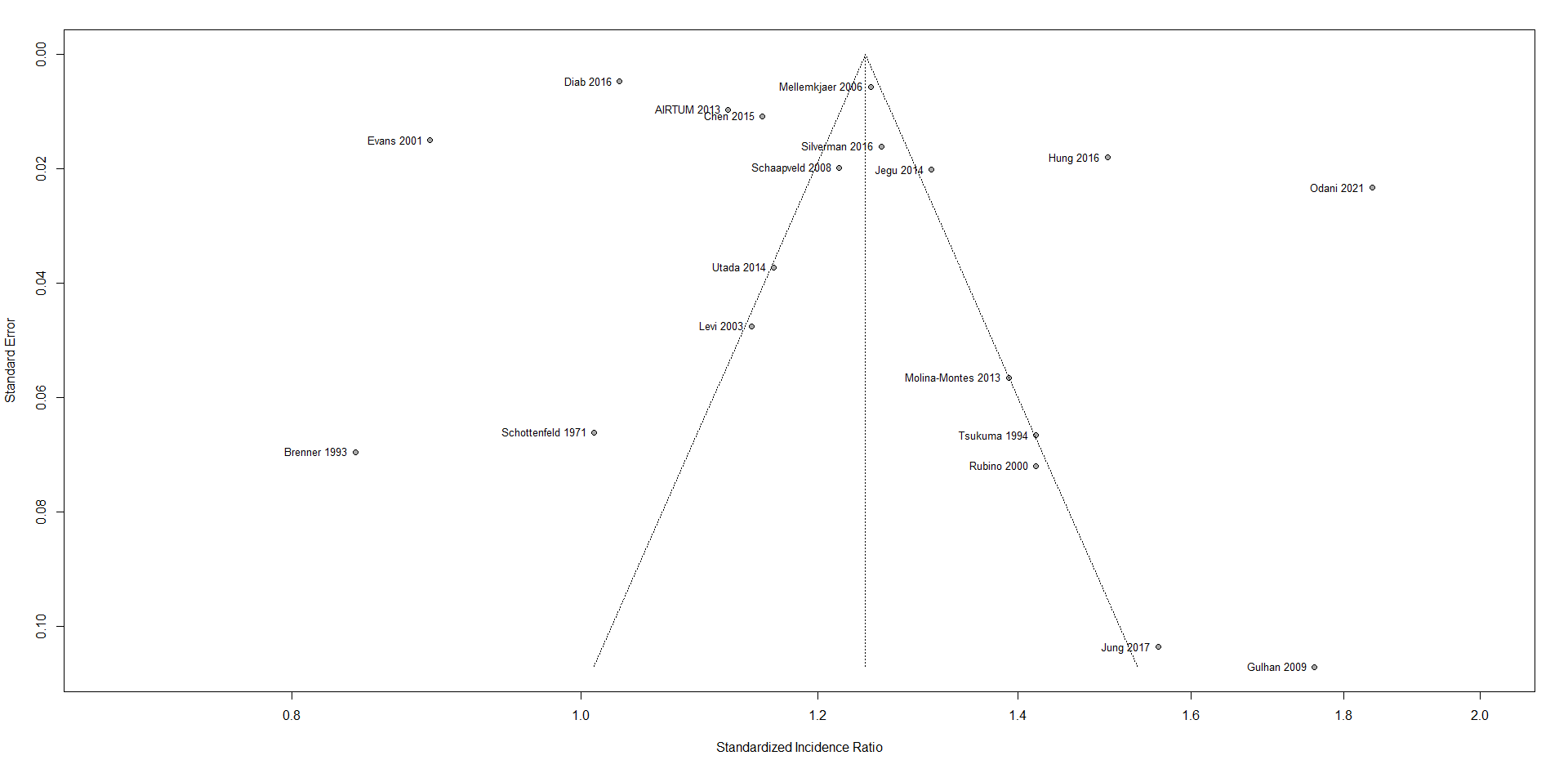
**Figure A1: Funnel plot: standard error against standardized incidence ratio among studies included in the unstratified meta-analysis.**

**Figure A2: Second non-breast primary risks following first primary breast cancers. Stratification: follow-up time duration (5 years).**


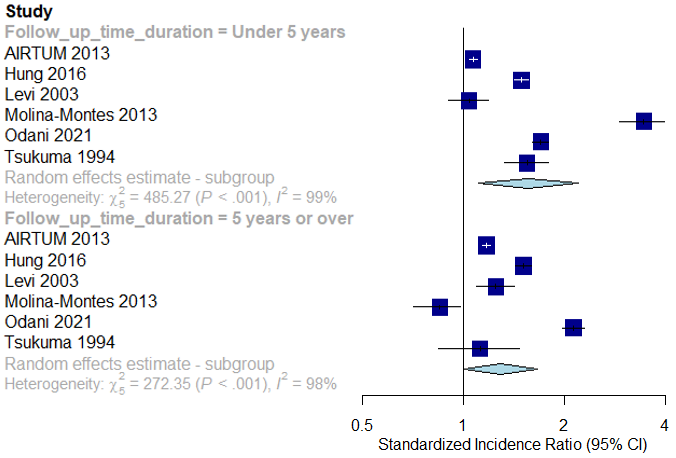


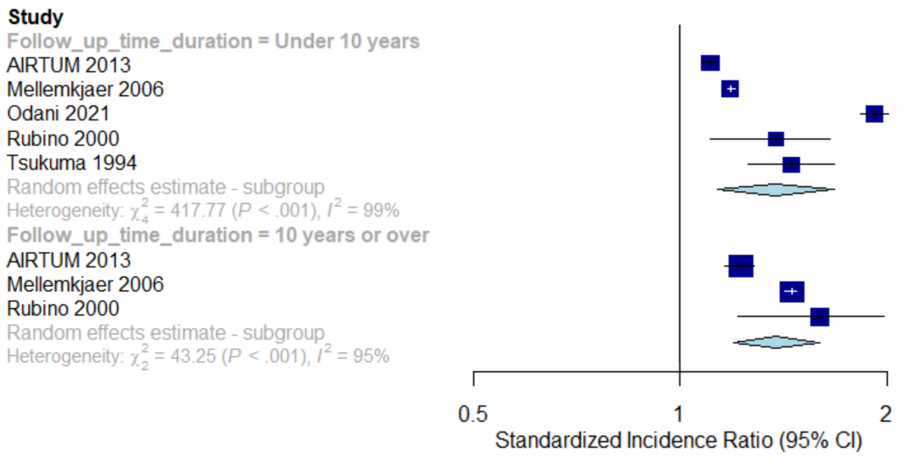
**Figure A3: Second non-breast primary risks following first primary breast cancers. Stratification: follow-up time duration (10 years).**

**Table A1: Newcastle-Ottawa scale scores, assigned to each study in systematic review**

| **Author and publication year** | **NOS^1^ scale selection score (/4)** | **NOS scale comparability score (/2)** | **NOS scale outcome score (/3)** | **NOS scale total score (/9)** |
| --- | --- | --- | --- | --- |
| AIRTUM Working Group 2013 (1) | 4 | 2 | 2 | 8 |
| Andersson 2008 (2) | 2 | 2 | 2 | 6 |
| Brenner 1993 (3) | 4 | 2 | 1 | 7 |
| Brown 2007 (4) | 3 | 2 | 2 | 7 |
| Chen 2015 (5) | 4 | 2 | 1 | 7 |
| Diab 2016 (6) | 3 | 1 | 2 | 6 |
| Evans 2001 (7) | 4 | 2 | 2 | 8 |
| Gulhan 2009 (18) | 3 | 2 | 1 | 6 |
| Harvey 1985 (8) | 4 | 2 | 2 | 8 |
| Hung 2016 (19) | 3 | 2 | 1 | 6 |
| Jégu 2014 (9) | 3 | 2 | 1 | 6 |
| Jung 2017 (20) | 2 | 0 | 2 | 4 |
| Lee 2008 (21) | 3 | 2 | 2 | 7 |
| Levi 2003 (10) | 4 | 2 | 2 | 8 |
| Mellemkjaer 2006 (33) | 4 | 2 | 2 | 8 |
| Molina-Montes 2013 (11) | 3 | 1 | 2 | 6 |
| Murakami 1987 (22) | 4 | 2 | 1 | 7 |
| Odani 2021 (23) | 4 | 1 | 1 | 6 |
| Ricceri 2015 (12) | 4 | 1 | 1 | 6 |
| Rubino 2000 (13) | 2 | 1 | 1 | 4 |
| Schaapveld 2008 (14) | 3 | 2 | 1 | 6 |
| Schottenfeld 1971 (15) | 2 | 2 | 2 | 6 |
| Silverman 2016 (24) | 4 | 1 | 2 | 7 |
| Tabuchi 2012 (25) | 3 | 1 | 2 | 6 |
| Trama 2022 (16) | 3 | 2 | 2 | 7 |
| Tsukuma 1994 (26) | 3 | 1 | 2 | 6 |
| Utada 2014 (27) | 4 | 2 | 2 | 8 |
| Zheng 2018 (17) | 4 | 0 | 2 | 6 |

^1^Newcastle-Ottawa Scale

*This review was unregistered. No protocol was prepared.*
